# Insights into the Allosteric Effect of SENP1 Q597A Mutation on the Hydrolytic Reaction of SUMO1 via an Integrated Computational Study

**DOI:** 10.3390/molecules27134149

**Published:** 2022-06-28

**Authors:** Mingfei Ji, Zongtao Chai, Jie Chen, Gang Li, Qiang Li, Miao Li, Yelei Ding, Shaoyong Lu, Guanqun Ju, Jianquan Hou

**Affiliations:** 1 Department of Urology, The First Affiliated Hospital of Soochow University, Suzhou 215006, China; jimingfei2014@163.com (M.J.); ligang_202206@163.com (G.L.); liqqli@163.com (Q.L.); urologistlee@163.com (M.L.); 2 Department of Urology, Second Affiliated Hospital of Navy Medical University, Shanghai 200433, China; rexcj8298@163.com (J.C.); dingyelei@163.com (Y.D.); 3 Department of Hepatic Surgery VI, Eastern Hepatobiliary Surgery Hospital, Navy Medical University, Shanghai 200433, China; runout@163.com; 4 Department of Bioinformatics and Medicinal Chemistry Center, School of Medicine, Shanghai Jiao Tong University, Shanghai 200025, China; 5 Department of Urology, Dushuhu Public Hospital Affiliated to Soochow University, Suzhou 215000, China

**Keywords:** SUMO, SENP1, molecular dynamics simulation, allostery, allosteric modulation

## Abstract

Small ubiquitin-related modifier (SUMO)-specific protease 1 (SENP1) is a cysteine protease that catalyzes the cleavage of the C-terminus of SUMO1 for the processing of SUMO precursors and deSUMOylation of target proteins. SENP1 is considered to be a promising target for the treatment of hepatocellular carcinoma (HCC) and prostate cancer. SENP1 Gln597 is located at the unstructured loop connecting the helices α4 to α5. The Q597A mutation of SENP1 allosterically disrupts the hydrolytic reaction of SUMO1 through an unknown mechanism. Here, extensive multiple replicates of microsecond molecular dynamics (MD) simulations, coupled with principal component analysis, dynamic cross-correlation analysis, community network analysis, and binding free energy calculations, were performed to elucidate the detailed mechanism. Our MD simulations showed that the Q597A mutation induced marked dynamic conformational changes in SENP1, especially in the unstructured loop connecting the helices α4 to α5 which the mutation site occupies. Moreover, the Q597A mutation caused conformational changes to catalytic Cys603 and His533 at the active site, which might impair the catalytic activity of SENP1 in processing SUMO1. Moreover, binding free energy calculations revealed that the Q597A mutation had a minor effect on the binding affinity of SUMO1 to SENP1. Together, these results may broaden our understanding of the allosteric modulation of the SENP1−SUMO1 complex.

## 1. Introduction

Small ubiquitin-related modifier (SUMO) modification, also called sumoylation, is a reversible process that catalyzes the post-translational modification of a target protein using a SUMO protein [1]. As an important mechanism in the regulation of numerous protein activities, sumoylation has been implicated in a number of biological processes, including signal transduction and cell proliferation, DNA replication and repair, cell cycle control, genome integrity, and protein localization [2,3]. Sumoylation is a highly dynamic process. The SUMO catalytic reaction is accomplished by E1, E2, and E3 ligases, while the reverse, desumolyation, is catalyzed by SUMO-specific proteases (SENPs) [4]. There are six SENP isoforms (SENP1–3 and SENP5–7) that have been identified in mammals; these are divided into three sub-families according to their sequence homology, subcellular localization, and substrate specificity [3]. SUMO proteins are expressed in their precursor forms, and SENPs catalyze the maturation process of SUMO proteins to expose their C-terminal Gly–Gly motif [1]. 

Among them, SENP1 has been associated with prostate tumorigenesis [5,6]. Accumulating evidence indicates that SENP1 is involved in the desumoylation of androgen receptor (AR), and that overexpression of SENP1 increases AR transcriptional activity, which has been found in >50% of high-grade precancerous prostate tissues, and in numerous prostate cancer cases [3,7]. Moreover, through comprehensive analysis of >150 specimens of prostate cancer, Wang et al. validated that there was a correlation between SENP1 expression and prostate cancer aggressiveness and recurrence [8]. Furthermore, based on quantitative PCR in human paired hepatocellular carcinoma (HCC), Cui et al. validated that deSUMOylation of hypoxia-inducible factor 1α (HIF-1α) by SENP1 is associated with increased cancer stemness in HCC and hepatocarcinogenesis under hypoxic conditions [9]. In addition, Tao et al. revealed that SENP1 is a crucial promotor for HCC through deSUMOylation of ubiquitin-conjugation enzyme E2T (UBE2T) [10]. Therefore, SENP1 is considered to be a promising target for the treatment of HCC and prostate cancer. 

**Figure 1:**
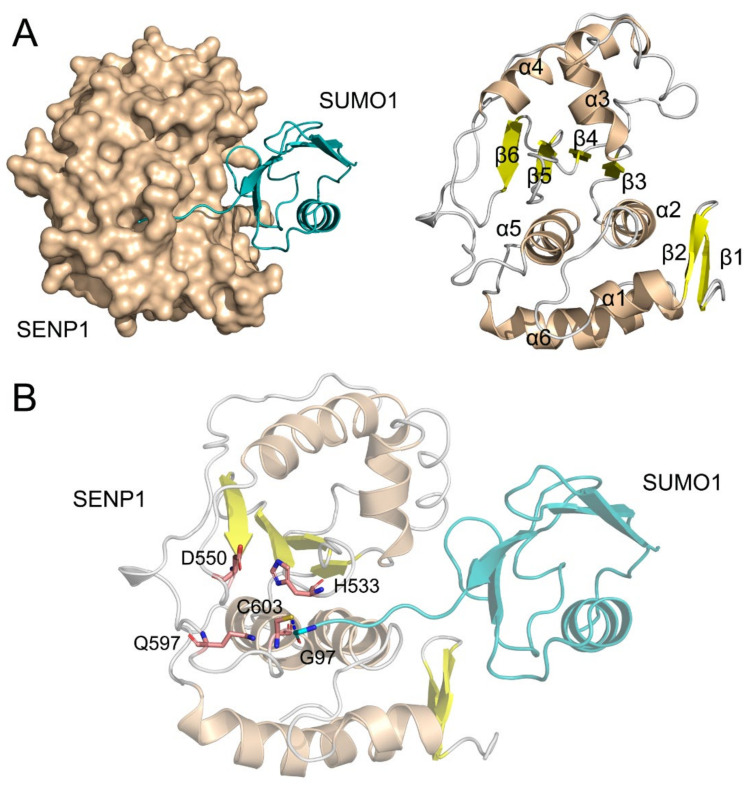
(**A**) Surface and cartoon representations of the C-terminal catalytic domain of SENP1 with SUMO1 (PDB ID: 2G4D). The secondary structural elements of α-helices, β-strands, and loops are colored using wheat, yellow, and gray, respectively; (**B**) Conformational rearrangement of the catalytic triad Cys603, His533, and Asp550 in the SENP1 active site. Cys603 of SENP1 forms a covalent thiohemiacetal linkage with the C-terminal carbonyl-C of Gly97 in SUMO1. Gln597 of SENP1 on the unstructured loop is shown as sticks.

Human SENP1 is a 644 amino acid protein which includes an N-terminal regulatory domain (residues 1–414) and a C-terminal catalytic domain (residues 415–643) [11,12]. The C-terminal domain controls catalytic activity, while the N-terminal domain modulates substrate specificity and cellular localization. To date, only crystal structures of the C-terminal catalytic domain of SENP1 have been solved in the apo form or with SUMO-bound protein–protein interactions (PPIs). The three-dimensional crystal structure of the C-terminal catalytic domain of SENP1 in complex with SUMO1 (residues 20–97) shows that the N-terminal subdomain of SENP1 contains two β-strands (β1 and β2) and four α-helices (α1, α2, α5, and α6), while the C-terminal subdomain includes a four stranded β-sheet (β3–β6) that is surrounded by two α-helices (α3 and α4) (Figure 1A). The C-terminal domain of SUMO1 forms an elongated strand that occupies the large cleft of SENP1. There is a covalent thiohemiacetal linkage between the active site Cys603 of SENP1 and the C-terminal carbonyl-C of Gly97 in SUMO1 (Figure 1B).

It should be noted that the protease catalytic triad Cys603, His533, and Asp550 are located in the SENP1 active site at the front of the helix α5, at the front of the β5, and at the end of the β6, respectively. The nucleophilic active site Cys603 is coordinated by the general base His533, which is in turn stabilized by Asp550 (Figure 1B). In vitro assays have demonstrated that each mutation of Cys603, His533, and Asp550 to alanine in SENP1 severely diminished its hydrolysis reaction with SUMO1 [3]. These results are explained based on the SENP1−SUMO1 structural complex, because the three residues form the catalytic center for the processing of pre-SUMO1 to its mature form. However, the Gln597 of SENP1 that is located at the unstructured loop is away from the interface with SUMO1, but the Q597A variant was severely impaired in the processing of SUMO1. Because Gln597 has no direct contact with the active sites of SENP1 and SUMO1, the effect of the Q597A mutation on the hydrolytic reaction of SUMO1 may be through an allosteric mechanism [13,14,15,16,17,18,19]. However, the underlying allosteric mechanism of the Q597A mutation in the disruption of SENP1 catalytic activity has remained unclear. 

Molecular dynamics (MD) simulations that capture the conformational dynamics of biomacromolecules are considered to be a well-established technique for exploring the conformational landscapes at an atomic level, and for directly uncovering biomolecular allosteric mechanisms [17,20,21,22,23,24,25]. Recently, we have employed MD simulations to probe the activation mechanism and conformational landscapes of G protein-coupled receptors (GPCRs) [26,27,28] and K-Ras4B [29,30,31,32], to elucidate the allosteric inhibition of CRISPR-Cas9 [33] and Argonaute−RNA complexes [34], and to discover cryptic allosteric sites in sirtuin 6 (SIRT6) [35,36,37], BCR-ABL1 [38], and epidermal growth factor receptor (EGFR) [39], as well as to decipher mutation-induced allosteric effects in PPIs [40,41,42].

Here, we employed multiple microsecond MD simulations of wild-type (WT) and Q597A SENP1−SUMO1 complexes to investigate SENP1 Q597A mutation-induced allosteric effects in the explicit water environment. Coupled with cross-correlation analysis, principal component analysis, and binding free energy calculations, we showed that the SENP1 Q597A mutation largely affects the arrangement of the catalytic Cys603 andHis533 in the active site, which might impair the catalytic activity of SENP1 to process SUMO1. The results obtained may broaden our understanding of the allosteric modulation of SENP1−SUMO1 complex.

## 2. Results and Discussion

### 2.1. System Stability

**Figure 2:**
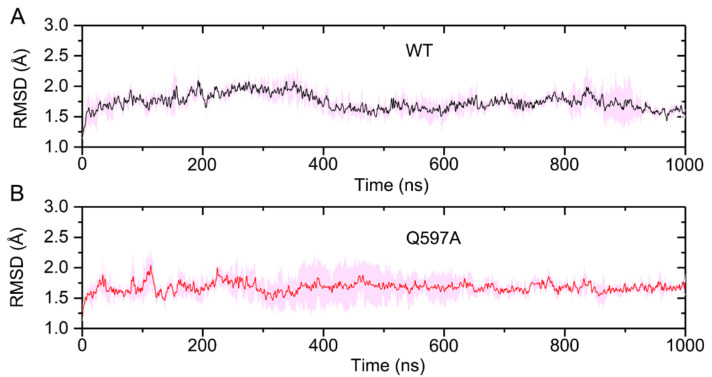
The root-mean-square deviation (RMSD) of all Cα atoms in the SENP1−SUMO1 complex for the WT (**A**) and the Q597A mutant; (**B**) averaged over three independent replicates. Transparent shading represents standard deviations.

To reveal the allosteric effect of SENP1 Q597A mutation on the hydrolysis of SUMO1, three replicate 1 μs MD simulations of WT and Q597A SENP1–SUMO1 complexes were performed. To unravel the conformational dynamics of the SENP1–SUMO1 complexes during the simulations, the root-mean-square deviation (RMSD) of all the Cα atoms was monitored for the three independent runs. As shown in Figure 2, the RMSD plots implied that both systems reached equilibrium after 400 ns of simulation, and the RMSD values for the WT and Q597A mutant were 1.74 ± 0.16 Å and 1.68 ± 0.20 Å, respectively. The mutant system had a similar RMSD value to the WT system, indicating that the single Q597A mutation on SENP1 had a minor effect on the overall stability of the protein complex. 

**Figure 3:**
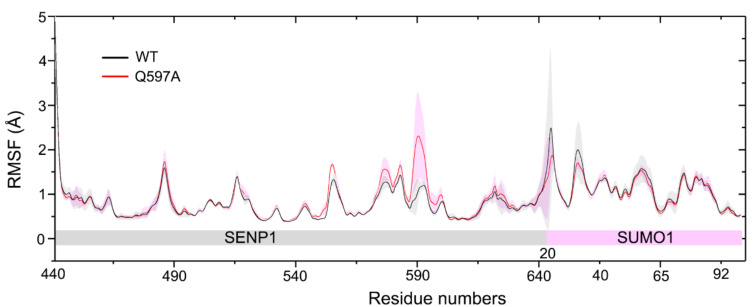
The root-mean-square fluctuation (RMSF) of all Cα atoms of the SENP1−SUMO1 complex for the WT and Q597A mutant systems. Average of three replicates is shown with standard deviation represented transparent shading.

To further illuminate the local conformational dynamics of SENP1–SUMO1 upon mutation, we calculated the atomic root-mean-square fluctuation (RMSF) for all Cα atoms in the three independent runs. As shown in Figure 3, compared to the WT system, a markedly higher RMSF was observed at the residues Ser571–Asp602 of SENP1 in the mutant system, which are located at the unstructured loop connecting the helices α4 to α5. Indeed, the SENP1 Q597A mutation site is located at this unstructured loop. This result suggested that the Q597A mutation had a significant effect on its nearby residues. As for SUMO1, the RMSF profiles in both the WT and mutant systems were similar, indicating that the SENP1 Q597A mutation had a subtle effect on the conformational dynamics of SUMO1. However, in comparing the conformational dynamics of the N- and C-terminals of SUMO1, we found that the C-terminus became more stable relative to the N-terminus, with the latter exhibiting a large conformational flexibility. This observation was reasonable, because in the crystal structure of the SENP1–SUMO1 complex, the C-terminal of SUMO1 inserts into the catalytic site of SENP1, forming strong contacts with SENP1 and stabilizing the C-terminal SUMO1; while the N-terminal of SUMO1 has no direct contacts with SENP1, thereby demonstrating the noticeable conformational dynamics of the N-terminal of SUMO1 during the simulations.

### 2.2. Q597A Mutation Enhanced the Coupled Motions of Protein Domains

**Figure 4:**
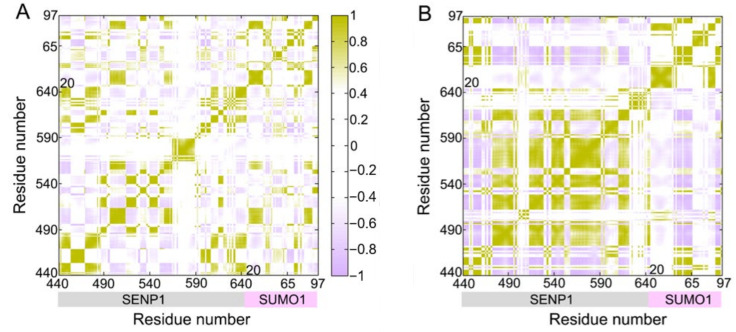
Dynamic cross-correlation matrix (CC*ij*) for the WT (**A**) and Q597A (**B**) mutant systems. The correlated motions are colored green-yellow (CC*ij* > 0), while the anti-correlated motions are colored purple (CC*ij* < 0). Color scales are shown to the right. The CC*ij* values with an absolute correlation coefficient of <0.4 are colored white for clarity.

Dynamic cross-correlation matrix (DCCM) analysis was carried out to probe the interdependent conformational motions of the SENP1–SUMO1 complex among spatially different domains in both the WT and Q597A mutant systems. In the DCCM calculations, the CCij matrix reflects the correlated motions between the two Cα atoms (i and j), representing whether they move as correlated motions (CCij > 0) or as anti-correlated motions (CCij < 0). As shown in Figure 4, the CCij matrix of the SENP1–SUMO1 complex showed a conserved pattern of correlated and anti-corelated motions in both the WT and Q597A mutant systems. However, in SENP1, the Q597A mutant system had a higher correlated motion in the region of residues ~Ile470–Ile620 compared to the WT system. This result was consistent with the RMSF analysis, in which the Q597A mutant system yielded an enhanced residue fluctuation in the region of SENP1 Ser571–Asp602 located at the unstructured loop connecting the helices α4 to α5. In contrast, the Q597A mutation caused a minor effect on the conformational dynamics of SUMO1. Collectively, these data indicated that the Q597A mutation yielded an enhancement of the correlated motions of protein domains in SENP1.

### 2.3. Pricipal Component Analysis (PCA)

In order to dissect the large-scale collective motions of the SENP1−SUMO1 complex and to reveal the effect of Q597A mutation on its conformational dynamics, principal component analysis (PCA) was carried out. According to PCA, the first two principal models of motion (i.e., principal components 1 and 2, PC1 and PC2) offer knowledge regarding the large amplitude motions of the SENP1−SUMO1 complex. During PCA, the three replicates of the simulated trajectories for both the WT and Q597A mutant systems were used, and subjected to RMS fitting to the same crystal structures of the SENP1−SUMO1 complex. This operation ensured the consistency of the PCs. 

**Figure 5:**
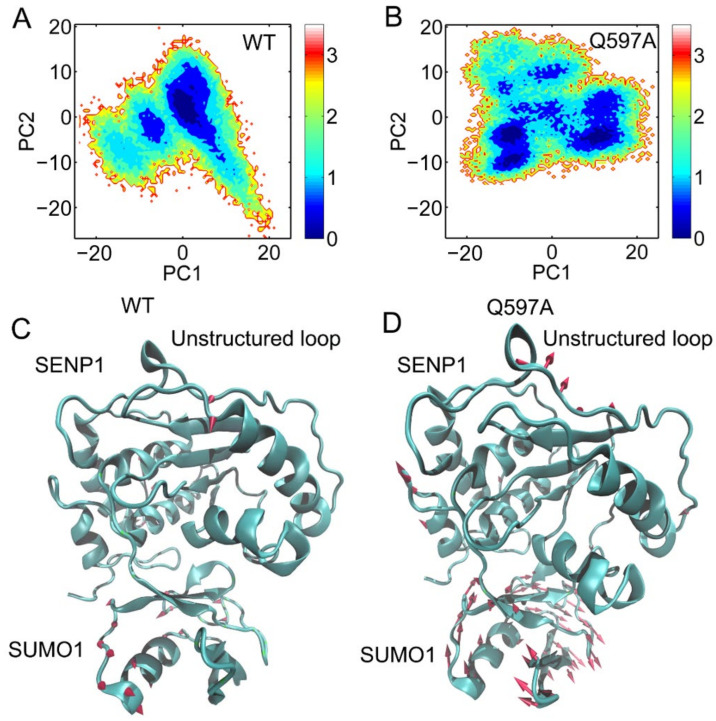
Projections of the first and second principal components (PC1 and PC2) from three replicate MD simulations for the WT (**A**) and Q597A mutant (**B**) systems. PC1 of the SENP1–SUMO1 complex for the WT (**C**) and Q597A mutant (**D**) systems. The sizes of the red arrows are proportional to the amplitude of motions.

First, PC1 and PC2 were plotted over the two-dimensional surfaces of the SENP1−SUMO1 complex in the WT and Q597A mutant systems. As a result, the PC1 vs. PC2 plots for the WT complex showed one major state (Figure 5A), while two states were observed in the Q597A mutant system (Figure 5B). This indicated that the Q597A mutation induced conformational changes in the SENP1−SUMO1 complex. To further reveal which protein domains were affected by the Q597A mutation, PC1 was plotted over the three-dimensional structures of the SENP1−SUMO1 complex in the WT and Q597A systems, using arrows to represent the direction and the relative amplitude of motions. As shown in Figure 5C, in the WT system, SUMO1 showed moderate amplitude motions, while SENP1 displayed no obvious motions. In contrast, in the Q597A mutant system (Figure 5D), SUMO1 showed large amplitude motions and SENP1 displayed obvious motions in the region of the unstructured loop connecting the helices α4 to α5. The PCA results unveiled an observation that the Q597A mutation induced marked motion of the unstructured loop in SENP1, which was agreement with the RMSF analysis. An increased flexibility of the unstructured loop in response to the Q597A mutation could have an essential role on the influence of the hydrolytic reaction of SUMO1. 

### 2.4. Community Network Analysis

**Figure 6:**
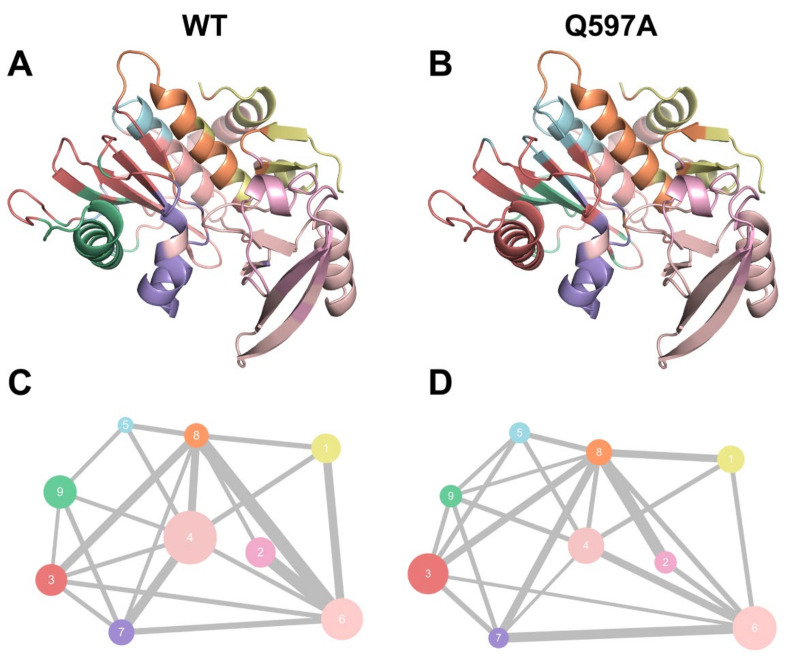
Colored community network of WT (**A**,**C**) and Q597A (**B**,**D**) systems. Each sphere represents an individual community with its size correlated with the number of residues.

To unveil the allosteric differences between the WT and Q597A SENP1−SUMO1 complexes, allosteric community analysis was carried out to calculate mutual information for all residues based on the Girvan–Newman algorithm. The analysis can assess the varied coupling among the different communities. In all simulated trajectories, the two Cα atoms of any residues within a cut-off distance of 4.5 Å keeping >75% of simulation time were deemed as belonging to the same community. The community was regarded as a coordinated unit within the overall protein structure. Visualization of the community network graphs can uncover the corresponding intensity of the different communities by the means of comparing the allosteric networks of the WT and Q597A systems within SENP1-SUMO1. Both WT (Figure 6A,C) and Q597A (Figure 6B,D) SENP1−SUMO1 complexes were divided into nine communities. Moreover, in both systems, the SUMO1 part largely belonged to Community 6, while the SENP1 part constituted Communities 1–5 and 7–9. These results suggested that the Q597A mutation did not change the number of communities within the SENP1–SUMO1 complex. However, the Q597A mutation induced the sizes and intensities of several communities to be changed. For example, the sizes of Communities 2, 4, 7, and 9 in the Q597A mutant were decreased compared to the WT, while the sizes of Communities 3 and 5 became larger in the Q597A mutant compared to those in the WT. In fact, the SENP1 residues Ser571–Asp602 were in Community 3 and the C-terminal helix α5 was in Community 5. The newly formed communication between Ser571–Asp602 and the C-terminal of helix α5 in the mutant would affect the conformational dynamics of the N-terminal helix α5 occupied by the catalytic Cys603. The three remaining communities, 1, 6, and 8, were unchanged in both the WT and Q597A systems. Indeed, the three communities 1, 6, and 8 mainly comprised the interface of SENP1−SUMO1 protein–protein interactions. These results implied that the Q597A mutation changes the communication network of SENP1, but may have a minor effect on SUMO1 dynamic communication.

### 2.5. Q597A Mutation Caused Conformational Changes to Cys603 and His533

**Figure 7:**
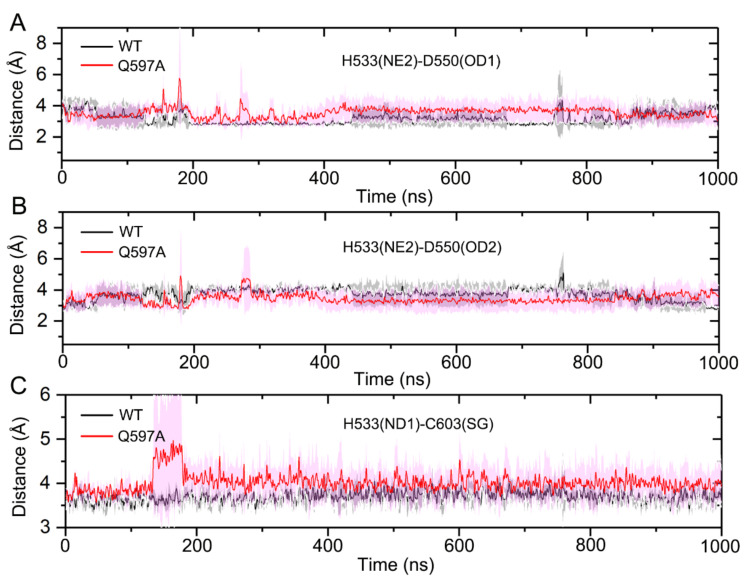
Time-dependence of the distances between the NE2 atom of His533 and the OD1 atom of Asp550 (**A**), the NE2 atom of His533 and the OD1 atom of Asp550 (**B**), and the ND1 atom of His533 and the SG atom of Cys603 (**C**) for three independent runs with standard deviation represented by transparent shading.

**Figure 8:**
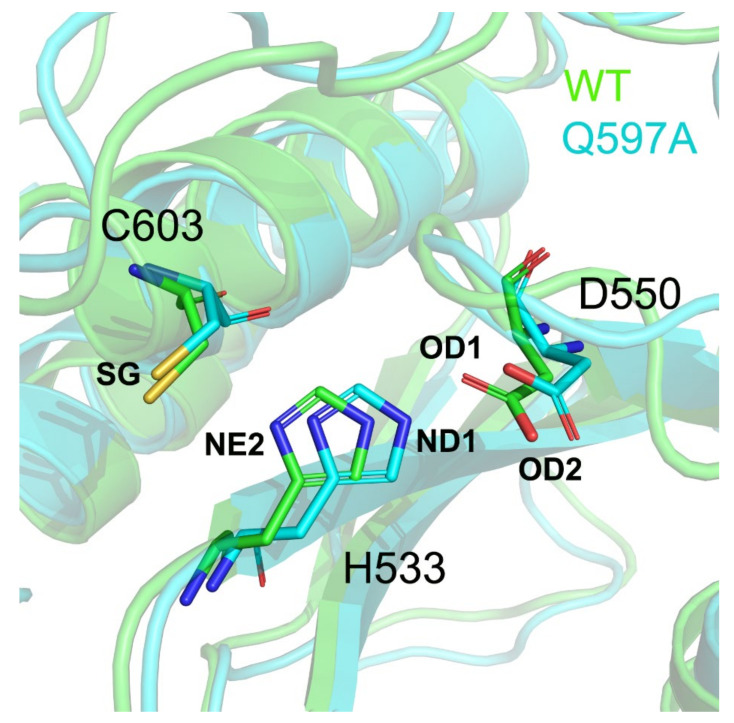
Structural superimposition of the SENP1 catalytic triad Cys603, His533, and Asp550 in the WT and Q597A systems.

In the SENP1 catalytic site, the catalytic triad Cys603, His533, and Asp550 forms direct interactions. His533 forms hydrogen bonding interactions with both Asp550 and Cys603. Thus, the correct arrangement of the catalytic triad in the SENP1 active site is critical for the hydrolysis of SUMO1. To reveal the impact of the Q597A mutation on the arrangement of the catalytic triad, we monitored the conformational dynamics of the catalytic triad during the simulations. We calculated the distances between the NE2 atom of His533 and the OD1 atom of Asp550 (Figure 7A), the NE2 atom of His533 and the OD2 atom of Asp550 (Figure 7B), and the ND1 atom of His533 and the SG atom of Cys603 (Figure 7C) for the three independent runs. As shown in Figure 7A, the distance between the NE2 atom of His533 and the OD1 atom of Asp550 was stable in the WT system (3.44 ± 0.70 Å), while in the Q597A mutant, this distance was larger (3.74 ± 0.54 Å) than that in the WT system. The distance between the NE2 atom of His533 and the OD2 atom of Asp550, however, was slightly shorter in the Q597A mutant (3.18 ± 0.45 Å) compared to the WT system (3.56 ± 0.71 Å) (Figure 7B). These results suggested concerted changes in His533 and Asp550 in both systems. Notably, the distance between the His533 and the catalytic residue Cys603 varied due to the Q597A mutation (Figure 7C). In the WT system, the calculated distance was 3.69 ± 0.23 Å, which was shorter than that in the Q597A mutant (4.02 ± 0.49 Å). The analysis of the arrangement of the catalytic triad in both the WT and the Q597A mutant suggested that the mutation mainly affected the conformational arrangement of Cys603 and His533 of SENP1, affecting the hydrolysis of SUMO1. To further elucidate the arrangement of the catalytic triad, we extracted the most representative structural complexes of SENP1–SUMO1 using cluster analysis, and we superimposed the two conformations, focusing on the catalytic site. The k-means algorithm was used for cluster analysis, which first generated seed points, and then all the data points were iterated and each was assigned to its closest seed point. As shown in Figure 8, compared to the WT system, the catalytic triad Cys603, His533, and Asp550 showed conformational changes in the Q597A mutant system. Taken together, these results indicated that the SENP1 Q597A mutation caused the conformational changes of Cys603 and His533, which would impair the hydrolytic activity of SUMO1. 

### 2.6. Q597A Mutation Had a Minor Effect on the Binding Affinity of SUMO1 

To further reveal the effect of Q597A mutation on the binding affinity of SUMO1 to SENP1, a MM–PBSA binding free energy calculation was performed; this has been proven successful in evaluating protein–ligand or protein–protein interactions in different systems [43,44]. The last 200 ns trajectory in all three replicates for both the WT and Q597A systems were selected for the MM–PBSA binding free energy calculation. As shown in Table 1, the gas phase (ΔE_gas_) free energy of the SENP1–SUMO1 complex in the WT and Q597A mutant systems was −331.65 ± 15.88 and −324.55 ± 16.38 kcal/mol, respectively. The solvation free energy (ΔG_solvation_) of the SENP1–SUMO1 complex in the WT and Q597A mutant systems was −224.89 ± 12.72 and −220.79 ± 12.82 kcal/mol, respectively. Overall, the binding free energy (ΔG_binding_) of the SENP1–SUMO1 complex in the WT and Q597A mutant systems was −106.76 ± 6.46 and −103.77 ± 7.06 kcal/mol, respectively. The results show that the binding potencies of the SENP1–SUMO1 complex in the WT and Q597A mutant systems were similar, suggesting that the SENP1 Q597A mutation had a minor impact on the binding affinity of SUMO1 to SENP1.

## 3. Conclusions

In the present study, MD simulations in combination with multiple analysis methods, including RMSD, RMSF, DCCM, PCA, MM-PBSA, and community network analyses, were performed to elucidate the allosteric effect of the SENP1 Q597A mutation on the hydrolytic reaction of SUMO1. RMSF analyses revealed that Q597A mutation caused enhanced conformational dynamics of the unstructured loop linking the helices α4 to α5 occupied by the mutation site. DCCM analyses were carried out to reveal correlated motions in the SENP1–SUMO1 complex. The results revealed that Q597A mutation enhanced the correlated motions of protein domains in SENP1, rather than in SUMO1. The visualized community network data showed the same number of communities in the allosteric crosstalk for both the WT and Q597A mutant systems. Structural analysis further exposed that Q597A mutation caused conformational changes to Cys603 and His533 in the SENP1 catalytic site. MM-PBSA calculation further implied that the binding affinity of SUMO1 to each of WT and Q597A SENP1 was similar. Together, these data suggested that the allosteric effect of the Q597A mutation was through the impacts on Cys603 and His533 in SENP1, rather than on SUMO1, which may deepen our insight into the allosteric modulation of the SENP1–SUMO1 complex.

## 4. Materials and Methods

### 4.1. Structural Preparation

The 2.8 Å X-ray crystal structure of the SENP1−SUMO1 complex was obtained from the RCSB Protein Data Bank (PDB) (PDB ID: 2G4D) [11]. In 2G4D, the active site Cys603 of SENP1 was mutated to Ser603. Therefore, we mutated Ser603 back to Cys603 using the Discovery Studio program, and we manually adjusted the sidechain of SENP1 Cys603 to form a covalent thiohemiacetal linkage with the carbonyl-C of Gly97 in SUMO1. To construct the mutant, the SENP1 Gln597 was mutated to alanine to represent the SENP1^Q597A^−SUMO1 complex.

### 4.2. MD Simulations

MD simulations of SENP1−SUMO1 complexes in the explicit water environment were performed using the AMBER 18 package [45]. The force field for the protein was treated using AMBER FF14SB [46], and the TIP3P force field was used for the water molecules [47]. The covalent bond between SENP1C603 and SUMO1G97 was defined using the antechamber module. Both the WT and Q597A SENP1−SUMO1 complexes were solvated by TIP3P water molecules in an octahedral box, with a minimum distance of 10 Å between any protein atom and the edge of the water box. To simulate physiological conditions, 0.15 mol/L NaCl was added to both systems.

A two-stage energy minimization approach was performed to optimize the initial complexes using previous protocols [48,49,50]. First, we applied minimization of water molecules and ions (Na^+^ and Cl^−^) with positional restraints on protein atoms via a harmonic potential with a force constant of 500 kcal mol^−1^ Å^−2^. This process was completed with 5000 steps of the steepest descent energy minimization, followed by 5000 steps of the conjugate gradient energy minimization. Then, we applied minimization of all the atoms in the system without any restraints. This process was completed with 10,000 steps of the steepest descent energy minimization, followed by 20,000 steps of the conjugate gradient energy minimization. After minimization, both systems were heated gradually from 0 to 300 K within 300 ps, followed by constant temperature equilibration at 300 K for 700 ps, with positional restraints on protein atoms via a harmonic potential with a force constant of 10 kcal mol^−1^ Å^−2^, using a canonical NVT ensemble. Finally, three independent runs of 1 μs MD simulations were simulated with random velocities for both systems under NPT ensemble and periodic boundary conditions. An integration step of 2 fs was set for the simulations. Langevin dynamics were used to maintain a temperature at 300 K with a collision frequency of 1 ps^−1^. The particle mesh Ewald method was used to compute long-range electrostatic interactions [51]. A cutoff of 10 Å was set to calculate van der Waals and electrostatic interactions. The SHAKE method was used to constrain all covalent bonds involving hydrogen atoms [52]. 

### 4.3. Principal Component Analysis (PCA)

Principal component analysis (PCA) was performed to analyze the cartesian coordinates of the Cα atoms of the SENP1−SUMO1 complexes [53,54,55]. All simulated snapshots were used to construct a covariance matrix C*ij*:

$$Cij=\langle \left(x_{i}-\langle x_{i}\rangle \right)\left(x_{j}-\langle x_{j}\rangle \right)\rangle$$

where *x_i_* is a cartesian coordinate of the *i*th Cα atom, and 〈*x_i_*〉 represents the time averaged over all the configurations selected in the simulation. Before PCA analysis, translational and rotational motions were excluded by overlaying the backbone atoms of SENP1−SUMO1 complexes on the reference crystal structure. 

Diagonalization of the covariance matrix *C* yields the eigenvalues λ*i* and the corresponding eigenvectors *Vi*, namely, the principal component (PC). *Vi* represent the directions in the multidimensional space that correspond to independent modes of atomic motion, while λ*i* represent their corresponding amplitudes. The projection Proj (*M*, PC_*i*_) of any structure (snapshot) *M* onto the *i*th PC was calculated as follows:

Proj[*M*, *Vi*] = *M*_α_ ⋅ *Vi*

where *M*_α_ are the Cα atoms of the SENP1−SUMO1 complexes after overlaying *M* on the reference crystal structure.

### 4.4. Dynamic Cross-Correlation Analysis

The correlated motions were explored in both systems by calculating the dynamic cross-correlation matrix of the Cα atoms, described by the index *c_ij_* as follows:

$$c\left(i,j\right)=\frac{<\Delta r_{i}\cdot \Delta r_{j}>}{\sqrt{<\Delta r_{i}{>}^{2}\cdot <\Delta r_{j}{>}^{2}}}$$

where the fluctuation $\Delta r_{i}$ of the Cα atom of the *i*th residue along the simulated trajectory is calculated with respect to the average structure. The correlation values of *c(i,j)* fall in the [–1, 1] range. Positively correlated residues move in the same direction, whereas negatively (anti-correlated) residues move in the opposite direction. Values of 0 suggest the uncorrelated motions of two residues.

### 4.5. MM–PBSA Binding Free Energy Calculations

The molecular mechanics Poisson–Boltzmann surface area (MM−PBSA) method [43,44,56,57,58,59,60] was performed to calculate the binding free energy between SENP1 and SUMO1. The binding free energy (ΔG_binding_) was shown as Equation (1):

ΔG_binding_ = ΔG_complex_ − (ΔG_SENP1_ + ΔG_SUMO1_) (1)

where ΔG_complex_, ΔG_SENP1_, and ΔG_SUMO1_ are the free energies of the complex, SENP1, and SUMO1, respectively. Equation (1) can be further expressed as a sum of the absolute free energy in the gas phase (ΔE_gas_), the solvation free energy (ΔG_solvation_), and the entropy term (TΔS) (Equation (2)). The conformational entropy (−TΔS) was not calculated owing to challenges and inaccuracy in the estimation of the conformational entropy for the protein–protein interactions [59].

ΔG_binding_ = ΔE_gas_ + ΔG_solvation_ + TΔS (2)

ΔE_gas_ includes the van der Waals energy (ΔE_vdW_), electrostatic energy (ΔE_ele_), and internal energies (ΔE_int_) in the gas phase (Equation (3)):

ΔE_gas_ = ΔE_vdW_ + ΔE_ele_ + E_int_ (3)

ΔG_solvation_ can be calculated using Equation (4), including the polar contribution (ΔG_PB_) and the nonpolar contribution (ΔG_nonpolar_):

ΔG_solvation_ = ΔG_PB_ + ΔG_nonpolar_ (4)

ΔG_PB_ was calculated using the PB model. The ΔGnonpolar was calculated using Equation (5):

ΔG_nonpolar_ = γSASA + b (5)

where SASA is the solvent accessible surface area, with γ = 0.00542 kcal/(mol·Å^2^) and b = 0.92 kcal/mol, respectively.

### 4.6. Community Network Analysis

The community network analysis was calculated based on the correlation coefficient matrix, Cij, through the NetworkView plugin for VMD [55]. The Cα atoms of each residue in the SENP1−SUMO1 complex were considered to be a group of nodes that were connected by edges. The edges in the dynamic network were weighted according to the correlation data obtained from the DCCM results. The formation of the edges was calculated between the two nodes within a cut-off distance of 4.5 Å for >75% of the total of simulation time. We calculated the edge connections between two nodes using Equation (6):

d_*i,j*_ = −log(|C*i*,*j*|) (6)

where *i* and *j* represent two nodes, and C*ij* was calculated from the dynamical cross-correlation matrix of the Cα atoms.

## Figures and Tables

**Table 1:** Binding free energy (kcal/mol) between SENP1 and SUMO1 in the WT and Q597A systems.

	WT	Q597A
ΔG_gas_	−331.65 ± 15.88	−324.55 ± 16.38
ΔG_solvation_	224.89 ± 12.72	220.79 ± 12.82
ΔG_binding_	−106.76 ± 6.46	−103.77 ± 7.06

## Data Availability

Samples of the simulation trajectories are available from the authors upon request.

## References

[1] Mukhopadhyay D., Dasso M. (2007). Modification in reverse: The SUMO proteases. Trends Biochem. Sci..

[2] Yamaguchi T., Sharma P., Athanasiou M., Kumar A., Yamada S., Kuehn M.R. (2005). Mutation of SENP1/SuPr-2 Reveals an Essential Role for Desumoylation in Mouse Development. Mol. Cell. Biol..

[3] Kumar A., Zhang K.Y.J. (2015). Advances in the development of SUMO specific protease (SENP) inhibitors. Comput. Struct. Biotechnol. J..

[4] Van Nguyen T., Angkasekwinai P., Dou H., Lin F.M., Lu L.S., Cheng J., Chin Y.E., Dong C., Yeh E.T.H. (2012). SUMO-Specific Protease 1 Is Critical for Early Lymphoid Development through Regulation of STAT5 Activation. Mol. Cell.

[5] Cheng J., Bawa T., Lee P., Gong L., Yeh E.T.H. (2006). Role of desumoylation in the development of prostate cancer. Neoplasia.

[6] Zuo Y., Cheng J.K. (2009). Small ubiquitin-like modifier protein-specific protease 1 and prostate cancer. Asian J. Androl..

[7] Kaikkonen S., Jääskeläinen T., Karvonen U., Rytinki M.M., Makkonen H., Gioeli D., Paschal B.M., Palvimo J.J. (2009). SUMO-specific protease 1 (SENP1) reverses the hormone-augmented SUMOylation of androgen receptor and modulates gene responses in prostate cancer cells. Mol. Endocrinol..

[8] Wang Q., Xia N., Li T., Xu Y., Zou Y., Zuo Y., Fan Q., Bawa-Khalfe T., Yeh E.T.H., Cheng J. (2013). SUMO-specific protease 1 promotes prostate cancer progression and metastasis. Oncogene.

[9] Cui C.P., Wong C.C.L., Kai A.K.L., Ho D.W.H., Lau E.Y.T., Tsui Y.M., Chan L.K., Cheung T.T., Chok K.S.H., Chan A.C.Y. (2017). SENP1 promotes hypoxia-induced cancer stemness by HIF-1α deSUMOylation and SENP1/HIF-1α positive feedback loop. Gut.

[10] Tao Y., Li R., Shen C., Li J., Zhang Q., Ma Z., Wang F., Wang Z. (2020). SENP1 is a crucial promotor for hepatocellular carcinoma through deSUMOylation of UBE2T. Aging.

[11] Xu Z., Chau S.F., Lam K.H., Chan H.Y., Ng T.B., Au S.W.N. (2006). Crystal structure of the SENP1 mutant C603S-SUMO complex reveals the hydrolytic mechanism of SUMO-specific protease. Biochem. J..

[12] Shen L.N., Dong C., Liu H., Naismith J.H., Hay R.T. (2006). The structure of SENP1-SUMO-2 complex suggests a structural basis for discrimination between SUMO paralogues during processing. Biochem. J..

[13] Lu S., Shen Q., Zhang J., Shen Q., Zhang J. (2019). Allosteric Methods and Their Applications: Facilitating the Discovery of Allosteric Drugs and the Investigation of Allosteric Mechanisms. Acc. Chem. Res..

[14] Lu S., Ji M., Ni D., Zhang J. (2018). Discovery of hidden allosteric sites as novel targets for allosteric drug design. Drug Discov. Today.

[15] Lu S., He X., Ni D., Zhang J. (2019). Allosteric Modulator Discovery: From Serendipity to Structure-Based Design. J. Med. Chem..

[16] Chen X., Li C., Wang D., Chen Y., Zhang N. (2020). Recent Advances in the Discovery of CK2 Allosteric Inhibitors: From Traditional Screening to Structure-Based Design. Molecules.

[17] Maloney R.C., Zhang M., Jang H., Nussinov R. (2021). The mechanism of activation of monomeric B-Raf V600E. Comput. Struct. Biotechnol. J..

[18] Foutch D., Pham B., Shen T. (2021). Protein conformational switch discerned via network centrality properties. Comput. Struct. Biotechnol. J..

[19] Nussinov R., Tsai C.-J. (2013). Allostery in disease and in drug discovery. Cell.

[20] Okeke C.J., Musyoka T.M., Sheik Amamuddy O., Barozi V., Tastan Bishop Ö. (2021). Allosteric pockets and dynamic residue network hubs of falcipain 2 in mutations including those linked to artemisinin resistance. Comput. Struct. Biotechnol. J..

[21] Hernández-Alvarez L., Oliveira A.B., Hernández-González J.E., Chahine J., Pascutti P.G., de Araujo A.S., de Souza F.P. (2021). Computational study on the allosteric mechanism of Leishmania major IF4E-1 by 4E-interacting protein-1: Unravelling the determinants of m7GTP cap recognition. Comput. Struct. Biotechnol. J..

[22] Zhang M., Jang H., Nussinov R. (2019). The mechanism of PI3Kα activation at the atomic level. Chem. Sci..

[23] An X., Bai Q., Bing Z., Liu H., Yao X. (2021). Insights into the molecular mechanism of positive cooperativity between partial agonist MK-8666 and full allosteric agonist AP8 of hGPR40 by Gaussian accelerated molecular dynamics (GaMD) simulations. Comput. Struct. Biotechnol. J..

[24] Han D., Wang H., Wujieti B., Zhang B., Cui W., Chen B.Z. (2021). Insight into the drug resistance mechanisms of GS-9669 caused by mutations of HCV NS5B polymerase via molecular simulation. Comput. Struct. Biotechnol. J..

[25] Hu X., Pang J., Zhang J., Shen C., Chai X., Wang E., Chen H., Wang X., Duan M., Fu W. (2022). Discovery of Novel GR Ligands toward Druggable GR Antagonist Conformations Identified by MD Simulations and Markov State Model Analysis. Adv. Sci..

[26] Lu S., He X., Yang Z., Chai Z., Zhou S., Wang J., Rehman A.U., Ni D., Pu J., Sun J. (2021). Activation pathway of a G protein-coupled receptor uncovers conformational intermediates as targets for allosteric drug design. Nat. Commun..

[27] Lu S., Zhang J. (2019). Small Molecule Allosteric Modulators of G-Protein-Coupled Receptors: Drug–Target Interactions. J. Med. Chem..

[28] Wang Y., Li M., Liang W., Shi X., Fan J., Kong R., Liu Y., Zhang J., Chen T., Lu S. (2022). Delineating the activation mechanism and conformational landscape of a class B G protein-coupled receptor glucagon receptor. Comput. Struct. Biotechnol. J..

[29] Lu S., Ni D., Wang C., He X., Lin H., Wang Z., Zhang J. (2019). Deactivation Pathway of Ras GTPase Underlies Conformational Substates as Targets for Drug Design. ACS Catal..

[30] Li X., Dai J., Ni D., He X., Zhang H., Zhang J., Fu Q., Liu Y., Lu S. (2020). Insight into the mechanism of allosteric activation of PI3Kα by oncoprotein K-Ras4B. Int. J. Biol. Macromol..

[31] Wang Y., Ji D., Lei C., Chen Y., Qiu Y., Li X., Li M., Ni D., Pu J., Zhang J. (2021). Mechanistic insights into the effect of phosphorylation on Ras conformational dynamics and its interactions with cell signaling proteins. Comput. Struct. Biotechnol. J..

[32] He X., Du K., Yuanhao W., Li M., Fan J., Ni D., Lu S., Biao X., Liu Y. (2022). Autopromotion of K-Ras4B feedback activation through an SOS-mediated long-range allosteric effect. Front. Mol. Biosci..

[33] Li X., Wang C., Peng T., Chai Z., Ni D., Liu Y., Zhang J., Chen T., Lu S. (2021). Atomic-scale insights into allosteric inhibition and evolutional rescue mechanism of Streptococcus thermophilus Cas9 by the anti-CRISPR protein AcrIIA6. Comput. Struct. Biotechnol. J..

[34] Zhuang H., Fan X., Ji D., Wang Y., Fan J., Li M., Ni D., Lu S., Li X., Chai Z. (2022). Elucidation of the conformational dynamics and assembly of Argonaute−RNA complexes by distinct yet coordinated actions of the supplementary microRNA. Comput. Struct. Biotechnol. J..

[35] Ni D., Wei J., He X., Rehman A.U., Li X., Qiu Y., Pu J., Lu S., Zhang J. (2021). Discovery of cryptic allosteric sites using reversed allosteric communication by a combined computational and experimental strategy. Chem. Sci..

[36] Lu S., Chen Y., Wei J., Zhao M., Ni D., He X., Zhang J. (2021). Mechanism of allosteric activation of SIRT6 revealed by the action of rationally designed activators. Acta Pharm. Sin. B.

[37] Zhang Q., Chen Y., Ni D., Huang Z., Wei J., Feng L., Su J.C., Wei Y., Ning S., Yang X. (2022). Targeting a cryptic allosteric site of SIRT6 with small-molecule inhibitors that inhibit the migration of pancreatic cancer cells. Acta Pharm. Sin. B.

[38] Zhang H., Zhu M., Li M., Ni D., Wang Y., Deng L., Du K., Lu S., Shi H., Cai C. (2022). Mechanistic Insights Into Co-Administration of Allosteric and Orthosteric Drugs to Overcome Drug-Resistance in T315I BCR-ABL1. Front. Pharmacol..

[39] Qiu Y., Yin X., Li X., Wang Y., Fu Q., Huang R., Lu S. (2021). Untangling Dual-Targeting Therapeutic Mechanism of Epidermal Growth Factor Receptor (EGFR) Based on Reversed Allosteric Communication. Pharmaceutics.

[40] Ni D., Li Y., Qiu Y., Pu J., Lu S., Zhang J. (2020). Combining Allosteric and Orthosteric Drugs to Overcome Drug Resistance. Trends Pharmacol. Sci..

[41] Lu S., Qiu Y., Ni D., He X., Pu J., Zhang J. (2020). Emergence of allosteric drug- resistance mutations: New challenges for allosteric drug discovery. Drug Discov. Today.

[42] Feng L., Lu S., Zheng Z., Chen Y., Zhao Y., Song K., Xue H., Jin L., Li Y., Huang C. (2021). Identification of an allosteric hotspot for additive activation of PPARγ in antidiabetic effects. Sci. Bull..

[43] Khan A., Ahsan O., Wei D.-Q., Ansari J.K., Najmi M.H., Muhammad K., Waheed Y. (2021). Computational Evaluation of Abrogation of HBx-Bcl-xL Complex with High-Affinity Carbon Nanotubes (Fullerene) to Halt the Hepatitis B Virus Replication. Molecules.

[44] Ramesh P., Shin W.H., Veerappapillai S. (2021). Discovery of a potent candidate for ret-specific non-small-cell lung cancer—a combined in silico and in vitro strategy. Pharmaceutics.

[45] Case D.A., Cheatham T.E., Darden T., Gohlke H., Luo R., Merz K.M., Onufriev A., Simmerling C., Wang B., Woods R.J. (2005). The Amber biomolecular simulation programs. J. Comput. Chem..

[46] Maier J.A., Martinez C., Kasavajhala K., Wickstrom L., Hauser K.E., Simmerling C. (2015). ff14SB: Improving the Accuracy of Protein Side Chain and Backbone Parameters from ff99SB. J. Chem. Theory Comput..

[47] Jorgensen W.L., Chandrasekhar J., Madura J.D., Impey R.W., Klein M.L. (1983). Comparison of simple potential functions for simulating liquid water. J. Chem. Phys..

[48] He X., Huang N., Qiu Y., Zhang J., Liu Y., Yin X.-L., Lu S. (2021). Conformational Selection Mechanism Provides Structural Insights into the Optimization of APC-Asef Inhibitors. Molecules.

[49] Li X., Qi Z., Ni D., Lu S., Chen L., Chen X. (2021). Markov State Models and Molecular Dynamics Simulations Provide Understanding of the Nucleotide-Dependent Dimerization-Based Activation of LRRK2 ROC Domain. Molecules.

[50] Liang S., Wang Q., Qi X., Liu Y., Li G., Lu S., Mou L., Chen X. (2021). Deciphering the Mechanism of Gilteritinib Overcoming Lorlatinib Resistance to the Double Mutant I1171N/F1174I in Anaplastic Lymphoma Kinase. Front. Cell Dev. Biol..

[51] Darden T., York D., Pedersen L. (1993). Particle mesh Ewald: An N.long(N)method for Ewald sums in large systems. J. Chem. Phys..

[52] Ryckaert J.-P., Ciccotti G., Berendsen H.J.C. (1977). Numerical integration of the Cartesian Equations of Motion of a System with Constraints: Molecular Dynamics of n-Alkanes. J. Comput. Phys..

[53] Li X., Ye M., Wang Y., Qiu M., Fu T., Zhang J., Zhou B., Lu S. (2020). How Parkinson’s disease-related mutations disrupt the dimerization of WD40 domain in LRRK2: A comparative molecular dynamics simulation study. Phys. Chem. Chem. Phys..

[54] Hyeon C., Jennings P.A., Adams J.A., Onuchic J.N. (2009). Ligand-induced global transitions in the catalytic domain of protein kinase A. Proc. Natl. Acad. Sci. USA.

[55] Sethi A., Eargle J., Black A.A., Luthey-Schulten Z. (2009). Dynamical networks in tRNA: Protein complexes. Proc. Natl. Acad. Sci. USA.

[56] Xie T., Yu J., Fu W., Wang Z., Xu L., Chang S., Wang E., Zhu F., Zeng S., Kang Y. (2019). Insight into the selective binding mechanism of DNMT1 and DNMT3A inhibitors: A molecular simulation study. Phys. Chem. Chem. Phys..

[57] Liu N., Zhou W., Guo Y., Wang J., Fu W., Sun H., Li D., Duan M., Hou T. (2018). Molecular Dynamics Simulations Revealed the Regulation of Ligands to the Interactions between Androgen Receptor and Its Coactivator. J. Chem. Inf. Model..

[58] Hou T., Wang J., Li Y., Wang W. (2011). Assessing the performance of the MM/PBSA and MM/GBSA methods. 1. The accuracy of binding free energy calculations based on molecular dynamics simulations. J. Chem. Inf. Model..

[59] Wang E., Sun H., Wang J., Wang Z., Liu H., Zhang J.Z.H., Hou T. (2019). End-Point Binding Free Energy Calculation with MM/PBSA and MM/GBSA: Strategies and Applications in Drug Design. Chem. Rev..

[60] Spratt A.N., Kannan S.R., Woods L.T., Weisman G.A., Quinn T.P., Lorson C.L., Sönnerborg A., Byrareddy S.N., Singh K. (2021). Evolution, correlation, structural impact and dynamics of emerging SARS-CoV-2 variants. Comput. Struct. Biotechnol. J..

